# Fabrication of full-color GaN-based light-emitting diodes on nearly lattice-matched flexible metal foils

**DOI:** 10.1038/s41598-017-02431-7

**Published:** 2017-05-18

**Authors:** Hyeryun Kim, Jitsuo Ohta, Kohei Ueno, Atsushi Kobayashi, Mari Morita, Yuki Tokumoto, Hiroshi Fujioka

**Affiliations:** 1 Institute of Industrial Science (IIS), The University of Tokyo, 4-6-1 Komaba, Tokyo, 153-8505 Japan; 2 PRESTO, Japan Science and Technology Agency, 4-1-8 Honcho, Kawaguchi Saitama, 332-0012 Japan; 3 ACCEL, Japan Science and Technology Agency, 5 Sanbancho, Chiyoda-ku Tokyo, 102–0075 Japan

## Abstract

GaN-based light-emitting diodes (LEDs) have been widely accepted as highly efficient solid-state light sources capable of replacing conventional incandescent and fluorescent lamps. However, their applications are limited to small devices because their fabrication process is expensive as it involves epitaxial growth of GaN by metal-organic chemical vapor deposition (MOCVD) on single crystalline sapphire wafers. If a low-cost epitaxial growth process such as sputtering on a metal foil can be used, it will be possible to fabricate large-area and flexible GaN-based light-emitting displays. Here we report preparation of GaN films on nearly lattice-matched flexible Hf foils using pulsed sputtering deposition (PSD) and demonstrate feasibility of fabricating full-color GaN-based LEDs. It was found that introduction of low-temperature (LT) grown layers suppressed the interfacial reaction between GaN and Hf, allowing the growth of high-quality GaN films on Hf foils. We fabricated blue, green, and red LEDs on Hf foils and confirmed their normal operation. The present results indicate that GaN films on Hf foils have potential applications in fabrication of future large-area flexible GaN-based optoelectronics.

## Introduction

GaN and the related group III nitrides are key materials in high-efficiency LEDs^1, 2^. Most of the commercially available GaN-based LEDs have been fabricated by MOCVD on single-crystalline sapphire wafers because of their high thermal and chemical stability^3^. However, applications of GaN-based LEDs are often restricted because the use of sapphire as the substrate for GaN epitaxy has significant problems of small area, high cost, and difficulty in processing. GaN on sapphire also suffers from large mismatches in lattice constants (16%) and thermal expansion coefficients (34%), which leads to the formation of high-density crystalline defects in GaN films. To address these issues and expand the application field of GaN-based LEDs, a technique to grow high-quality GaN films on alternative substrates needs to be developed^4–6^.

Metals have recently emerged as a promising substrate for this purpose^7–10^, since metal foils generally possess flexibility and high thermal and electrical conductivity, and large-area metal foils can be prepared by a rolling process at a reasonable cost. Among various metals, hafnium (Hf) is an ideal substrate material for GaN growth because it shares many similarities in structural properties with GaN, including a similar space symmetry group (P6_3_/mmc (Hf) and P6_3_mc (GaN)) and small mismatches in the *a-*axis lattice constant (0.3%) and the thermal expansion coefficient (5.3%) between GaN and Hf^9, 10^.

Despite these advantages, GaN growth on a Hf foil has not been practical because of two significant problems. One problem is the randomly oriented grains of commercially available Hf foils, which leads to poor crystalline quality of the overlaid GaN film. To solve this problem, a highly *c-*axis oriented Hf foil with a large grain size should be prepared before GaN growth. Annealing can be a simple approach to promote recrystallization of a metal foil, which can produce a highly oriented structure^11–13^. The other problem is the serious interfacial reactions between GaN and Hf during high-temperature growth in conventional techniques such as MOCVD^9, 10^. The interfacial reactions must be suppressed to grow high-quality GaN films on Hf foils. Recent progress in the epitaxial growth process based on PSD has made it possible to grow high-quality group III nitride epitaxial films even at room temperature (RT)^14–18^ because of the highly energetic group III atoms during PSD growth. Such LT growth can suppress the interfacial reactions between GaN and a chemically vulnerable substrate such as metals. In fact, LT epitaxial growth of GaN and AlN films on various single-crystalline metal substrates has been achieved^19, 20^. It should be noted that PSD is capable of industry-scale growth of GaN due to its high productivity and scalability. In this study, we investigated GaN growth on Hf foils by PSD and explored the feasibility of fabricating GaN-based full color LEDs on the Hf foils.

**Figure 1:**
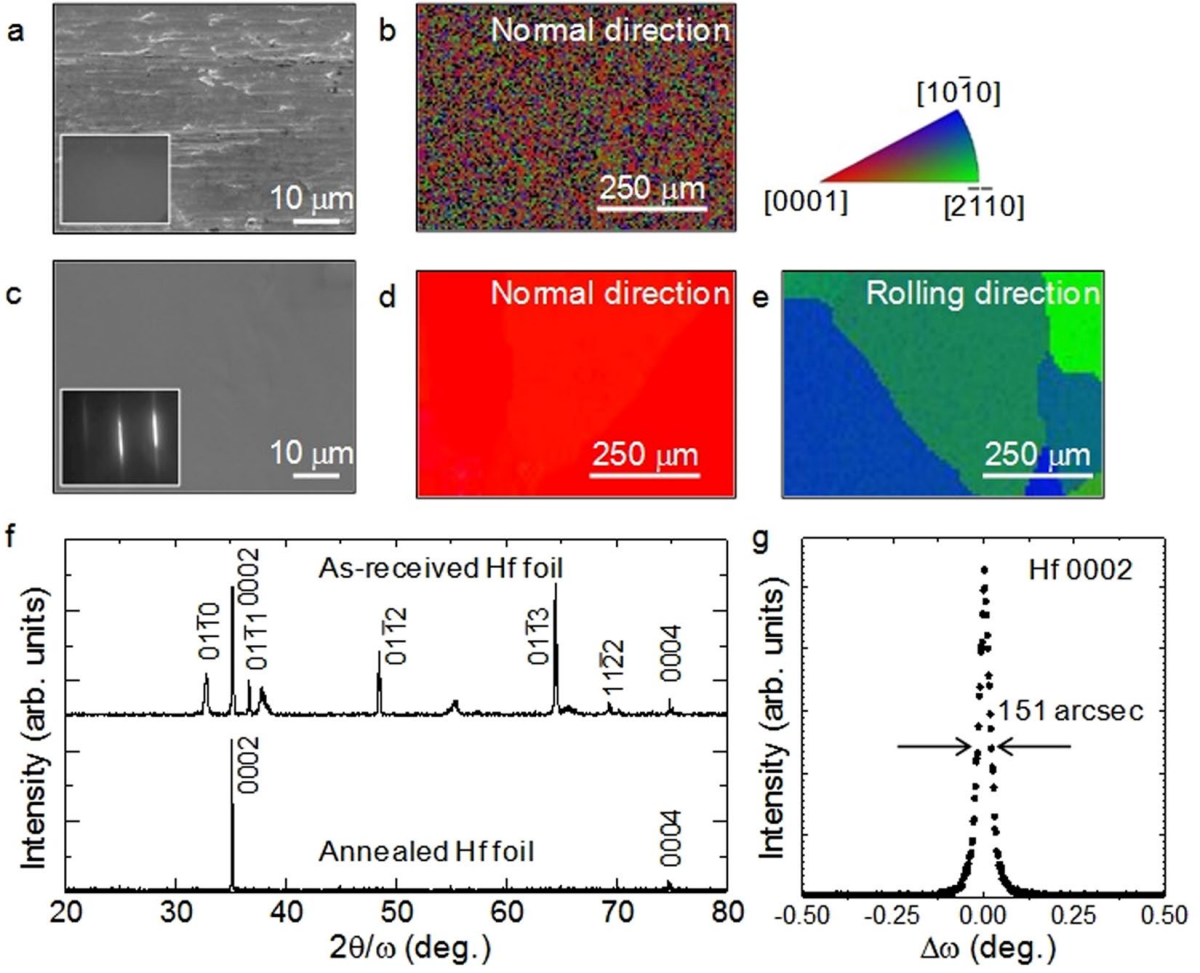
Surface structure and crystal orientations of Hf foils. (**a**) SEM image and (**b**) EBSD crystal orientation map along surface normal direction for the as-received Hf foil. (**c**) SEM image for the annealed Hf foil. (**d** and **e**) EBSD crystal orientation maps along the surface normal and rolling directions for the annealed Hf foil, respectively. Insets of (**a** and **c**) show RHEED patterns. (**f**) XRD curves of the as-received and annealed Hf foils. (**g**) XRC for the Hf 0002 diffraction of the annealed Hf foil.

A scanning electron microscope (SEM) image of an as-received 50-μm-thick Hf foil shows the foil surface to be rough (Fig. 1a), as expected from the rolling process for producing foils. The halo reflection high-energy electron diffraction (RHEED) pattern in the inset of Fig. 1a indicates that the surface is covered with an amorphous oxide layer. Figure 1b shows the crystal orientation map collected by electron backscattered diffraction (EBSD) in the normal direction of the surface. The Hf foil consists of randomly oriented grains with a size as small as 5 μm. To improve the crystalline quality and surface smoothness of Hf foils, we annealed the foils above 1000 °C in vacuum. After annealing, the surface smoothness was drastically improved, as seen in the SEM image of Fig. 1c. A sharp streaky diffraction pattern was seen in RHEED observations (inset of Fig. 1c), indicating removal of the amorphous oxide layer^10, 21^ and appearance of crystalline Hf with the smooth surface. Figure 1d shows the EBSD crystal orientation map along the surface normal direction. One can clearly see that the annealed Hf foils have highly *c-*axis oriented structure in the entire area. Also, the map in the rolling direction revealed that the grain size of the annealed *c-*axis oriented Hf foils was as large as 500 μm, as shown in Fig. 1e. X-ray diffraction (XRD) measurements were also performed to investigate the structural properties of the Hf foil before and after annealing. As shown in Fig. 1f, the as-received Hf foil showed multiple peaks indicating randomly oriented crystalline structures, while only {0001}-related diffraction peaks were observed for the annealed Hf foil. These XRD data are consistent to the EBSD results. The full width at half-maximum (FWHM) value of 0002 x-ray rocking curve (XRC) of the annealed Hf foil was as small as 151 arcsec, which is attributed to the highly *c-*axis orientation of the annealed Hf foil. These results indicate that the simple annealing process makes the Hf foils suitable for GaN crystalline growth.

**Figure 2:**
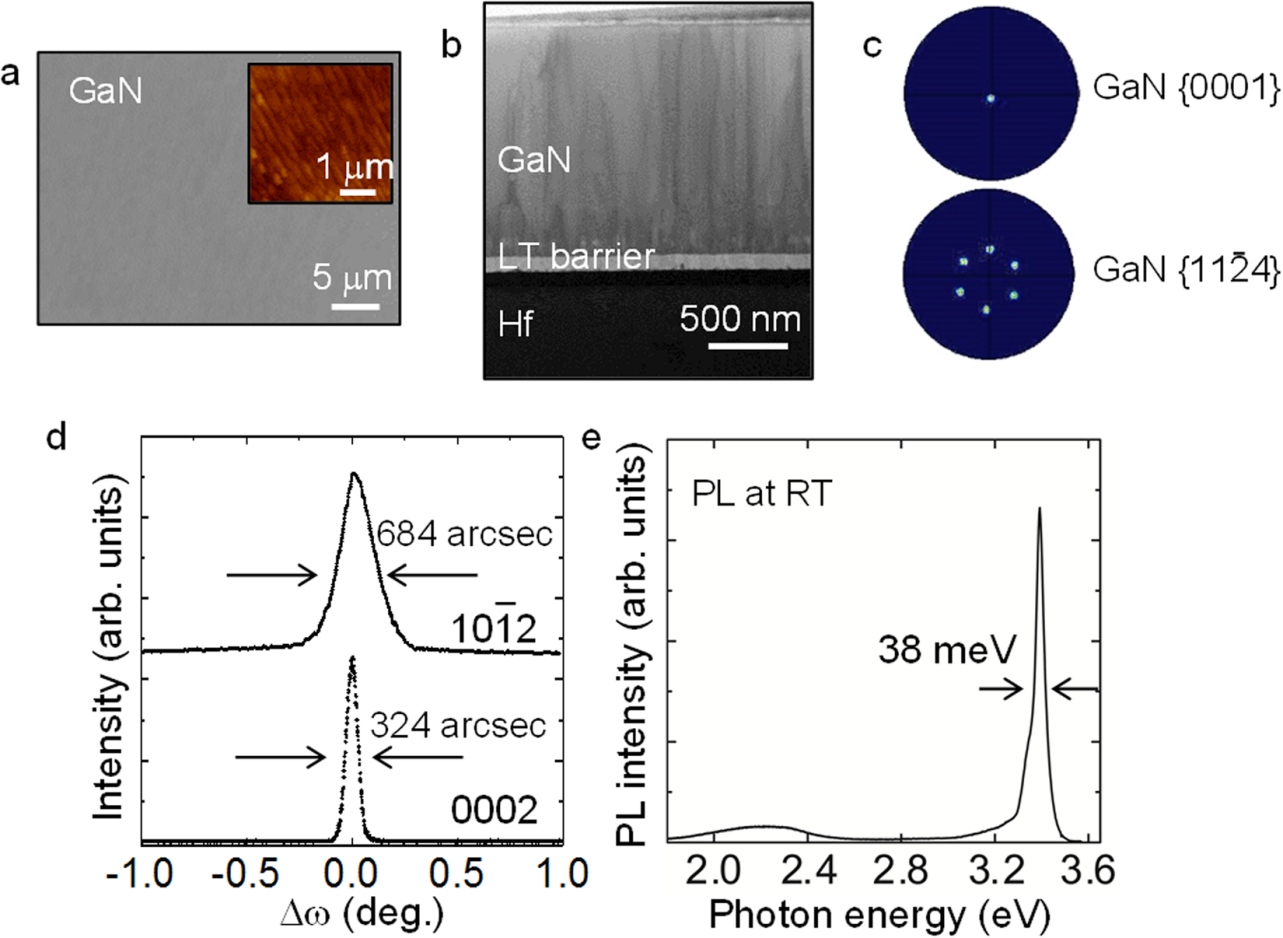
Structural and optical properties of GaN film on Hf foil with a LT-grown reaction barrier layer. (**a**) Surface SEM and (**b**) cross-sectional TEM images of the GaN film. Inset of (**a**) is a surface AFM image. (**c**) EBSD pole figures of {0001}_GaN_ and $\{11\overline{2}4\}$_GaN_. (**d**) 0002 and $10\overline{1}2$ XRCs, and (**e**) a RT-PL spectrum of the GaN film.

After the Hf foil was annealed, a 1-μm-thick GaN film was grown by PSD with a LT-grown reaction barrier layer. SEM observations showed that the GaN film surface is smooth (Fig. 2a), and atomic force microscope (AFM) observations revealed the surface has step-and-terrace structures with a root-mean-square (rms) value of 2.0 nm (inset of Fig. 2a), which indicates that GaN growth is two dimensional. Figure 2b illustrates the cross-sectional transmission electron microscope (TEM) image of the GaN film on the Hf foil. The heterointerface between GaN and Hf was smooth and sharp, indicating that introduction of the LT-grown reaction barrier layer significantly suppresses the interfacial reaction during GaN growth on Hf. Figure 2c shows EBSD pole figures for a 20 × 20 μm^2^ area of the GaN film. The {0001} spot for the GaN films was sharp and the $\{11\overline{2}4\}$ pole figure showed a clear six-fold rotational symmetry. This result indicates that the GaN film has a single domain structure, at least in the EBSD scanned area (20 × 20 μm^2^), due to the constraint from Hf atoms. To investigate the crystalline quality of the GaN film, XRC measurements were performed (Fig. 2d). The FWHM values of 0002 and $10\overline{1}2$ XRCs of the GaN film were 324 and 684 arcsec, respectively. It should be noted that these values are comparable to those on conventional substrates such as sapphire or Si. Figure 2e shows the photoluminescence (PL) spectrum of the GaN film at RT. The GaN film exhibited a sharp near-band-edge emission from a hexagonal phase at around 3.4 eV, with the FWHM value as small as 38 meV. From these results, we infer that the use of LT-growth by PSD enables the production of epitaxial GaN films on Hf foils without interfacial reactions, which can be potentially used for fabrication of optoelectronic devices

**Figure 3:**
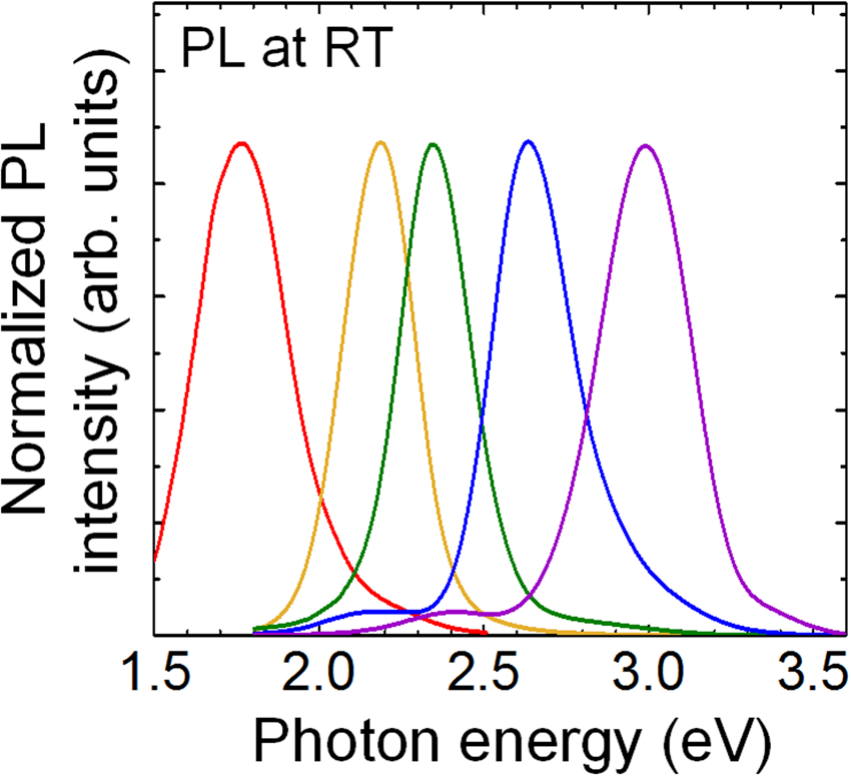
Optical properties of InGaN films. RT-PL spectra of InGaN films with various In compositions.

Then, we investigated InGaN growth on the Hf foils. An additional advantage in the use of LT growth based on the PSD technique is possible if we can grow InGaN films with high In compositions^16^, which is inherently important in construction of full-color LEDs. Figure 3 shows the RT-PL spectra for 20-nm-thick InGaN layers with various In compositions. The emission colors vary from violet (3.0 eV) to red (1.8 eV) with a change in In compositions

**Figure 4:**
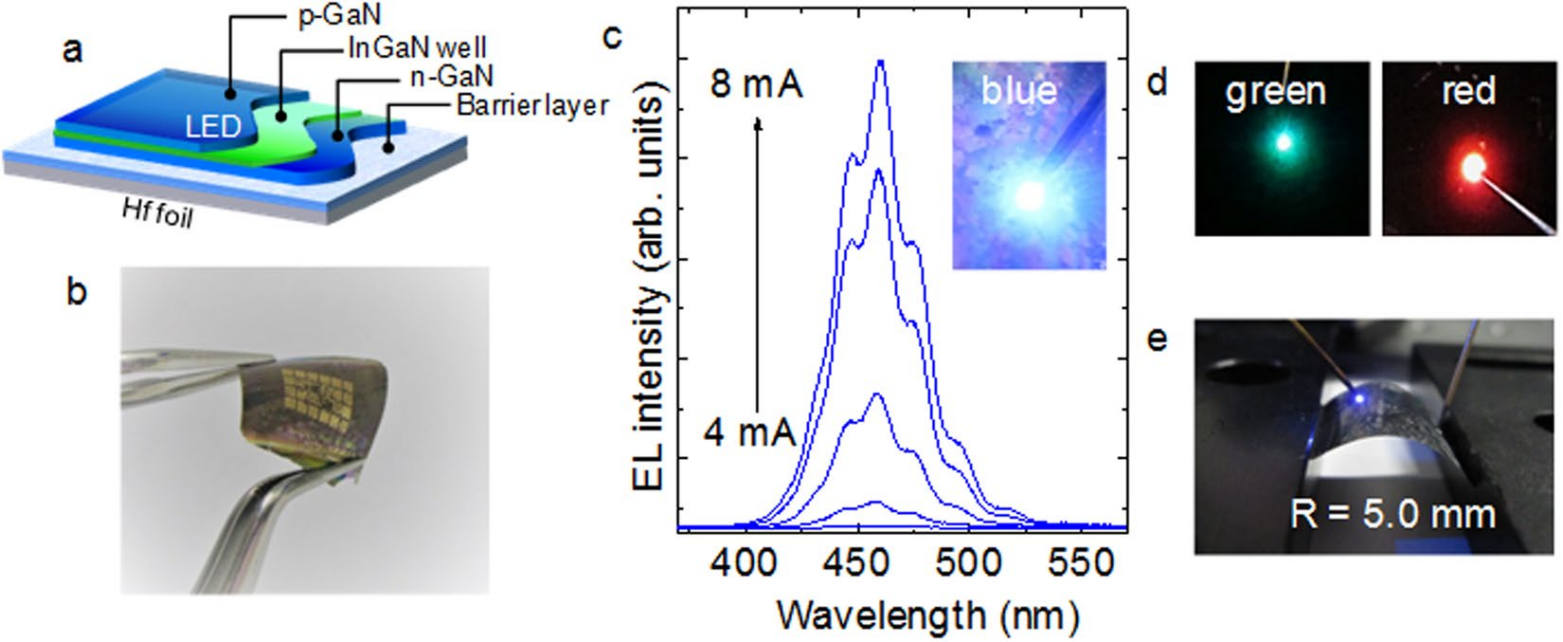
Fabrication of LEDs on Hf foils. (**a**) Schematic illustration of an LED structure. (**b**) Optical image of a flexible Hf foil with a GaN-based LED array. (**c**) EL spectra of the LED structure at forward currents ranging from 4 to 8 mA. The inset shows an optical image of blue EL at a forward current of 8 mA. (**d**) Photographs during the operation of green and red LEDs. (**e**) Light emission photograph at a bending radius of 5.0 mm.

To investigate the feasibility of device applications of the PSD-grown GaN and InGaN films prepared on Hf foils, we examined the operation of GaN-based LEDs fabricated on a Hf foil as a preliminary test. The LED structures were composed of p-GaN, InGaN multiple quantum wells (MQWs), and n-GaN. A schematic diagram and an optical image of an array of LEDs fabricated on a flexible Hf foil are shown in Fig. 4a and b, respectively. In current–voltage measurements, the LED structures exhibited good rectifying characteristics with a leakage current of 1 × 10^−4^ A at −5 V and a turn-on voltage of approximately 5 V. Figure 4c shows the electroluminescence (EL) spectra of the LEDs with various injection currents between 4 and 8 mA. The EL intensity of the blue light (approximately 460 nm) increased with an increase of the injection current, which indicates that blue LEDs can be fabricated and operated reasonably on flexible Hf foils. Green and red LEDs were also fabricated by altering the In composition in the InGaN wells (Fig. 4d), indicating the present technique enables the fabrication of full-color GaN-based LEDs on Hf foils. To test the LED operation on the Hf foil in a flexible form, the device was evaluated under substrate bending. Figure 4e shows the light emission photograph during a current injection of 5 mA at a bending radius of 5.0 mm. As shown in the photograph, the bended LED exhibited blue light emission without observable degradation.

These results indicate that GaN films on Hf foils have potential applications in future large-area GaN-based optoelectronic devices such as flexible displays.

## Methods

### Preparation of Hf foils

Commercially available 50-μm-thick Hf foils with 99.9% purity were used as substrates for GaN growth. After sonication in acetone for 10 min, the foils were annealed above 1000 °C for 60 min in vacuum to remove surface impurities such as oxygen and carbon and to produce the highly *c-*axis oriented structure.

### Growth and characterization of GaN films

The annealed Hf foils were introduced into a PSD chamber with a background pressure below 5 × 10^−10^ torr for nitride film growth. We prepared 1-µm-thick GaN films at 700 °C with LT-grown reaction barrier layers composed of 100-nm-thick AlN and 50-nm-thick HfN layers. The LT-growth temperature was set at around 400 °C. The growth rate of GaN was set at 1.0 µm/h. The sample surfaces were investigated by SEM, AFM, TEM, and RHEED. The structural properties of the GaN films were characterized by XRD using a Bruker D8 diffractometer and by EBSD using an INCA Crystal EBSD system connected to the SEM apparatus. The optical properties were investigated by PL measurements at RT with a He–Cd laser (λ = 325 nm) as the excitation source.

### LED fabrication

Five periods of InGaN (3 nm)/GaN (10 nm) MQWs were grown on a 1-μm-thick n-type GaN layer and topped by a 0.2-μm-thick Mg-doped p-type GaN layer. These layers were grown in a temperature range from 400 to 700 °C^16^. The Mg-doped GaN grown by PSD shows p-type conductivity without post annealing because the raw materials of the PSD growth system do not contain hydrogen atoms^16, 22^. In and Pd/Au electrodes were deposited on the n- and p-GaN layers, respectively, by e-beam evaporation to form ohmic contacts.

## References

[1] Akasaki I, Amano H (1997). Crystal growth and conductivity control of group III nitride semiconductors and their application to short wavelength light emitters. Jpn. J. Appl. Phys..

[2] Nakamura S (1998). The Roles of Structural Imperfections in InGaN-Based Blue Light-Emitting Diodes and Laser Diodes. Science.

[3] Liu L, Edgar JH (2002). Substrates for gallium nitride epitaxy. Mater. Sci. Eng. R.

[4] Chung K, Lee CH, Yi GC (2010). Transferable GaN layers grown on ZnO-coated graphene layers for optoelectronic devices. Science.

[5] Bour DP (2000). Polycrystalline nitride semiconductor light-emitting diodes fabricated on quartz substrates. Appl. Phys. Lett..

[6] Choi JH (2011). Nearly single-crystalline GaN light-emitting diodes on amorphous glass substrates. Nature Photonics.

[7] Freitas JA, Rowland LB, Kim J, Fatemi M (2007). Properties of epitaxial GaN on refractory metal substrates. Appl. Phys. Lett..

[8] Calabrese G (2016). Molecular beam epitaxy of single crystalline GaN nanowires on a flexible Ti foil. Appl. Phys. Lett..

[9] Beresford R, Paine DC, Briant CL (1997). Group IVB refractory metal crystals as lattice-matched substrates for growth of the group III nitrides by plasma-source molecular beam epitaxy. J. Cryst. Growth.

[10] Kim HR (2016). Epitaxial growth of GaN films on nearly lattice-matched hafnium substrates using a low-temperature growth technique. APL Materials.

[11] Seward GG, Celotto S, Prior DJ, Wheeler J, Pond RC (2004). *In situ* SEM-EBSD observations of the hcp to bcc phase transformation in commercially pure titanium. Acta Mater..

[12] Goussery V (2004). Grain size effects on the mechanical behavior of open-cell nickel foams. Adv. Eng. Mater..

[13] Al-Samman T, Gottstein G (2008). Dynamic recrystallization during high temperature deformation of magnesium. Mater. Sci. Eng. A.

[14] Sato K, Ohta J, Inoue S, Kobayashi A, Fujioka H (2009). Room-temperature epitaxial growth of high quality AlN on SiC by pulsed sputtering deposition. Appl. Phys. Express.

[15] Watanabe T (2014). AlGaN/GaN heterostructure prepared on a Si (110) substrate via pulsed sputtering. Appl. Phys. Lett..

[16] Nakamura E, Ueno K, Ohta J, Fujioka H, Oshima M (2014). Dramatic reduction in process temperature of InGaN-based light-emitting diodes by pulsed sputtering growth technique. Appl. Phys. Lett..

[17] Itoh T, Kobayashi A, Ueno K, Ohta J, Fujioka H (2016). Fabrication of InGaN Thin-Film Transistors using Pulsed Sputtering Deposition. Sci. Rep..

[18] Shon JW, Ohta J, Ueno K, Kobayashi A, Fujioka H (2014). Fabrication of full-color InGaN-based light-emitting diodes on amorphous substrates by pulsed sputtering. Sci. Rep..

[19] Okamoto K, Inoue S, Nakano T, Ohta J, Fujioka H (2009). Epitaxial growth of GaN on single-crystal Mo substrates using HfN buffer layers. J. Cryst. Growth.

[20] Inoue S, Okamoto K, Nakano T, Ohta J, Fujioka H (2007). Epitaxial growth of AlN films on Rh ultraviolet mirrors. Appl. Phys. Lett..

[21] Panish MB, Reif L (1963). Thermodynamics of the vaporization of Hf and HfO_2_: Dissociation energy of HfO. J. Chem. Phys..

[22] Arakawa Y, Ueno K, Kobayashi A, Ohta J, Fujioka H (2016). High hole mobility p-type GaN with low residual hydrogen concentration prepared by pulsed sputtering. APL Mater..

